# Effect of Carbon Nanotube and Styrene-Acrylic Emulsion Additives on Microstructure and Mechanical Characteristics of Cement Paste

**DOI:** 10.3390/ma13122807

**Published:** 2020-06-22

**Authors:** Jie Fan, Gengying Li, Sijie Deng, Chengwei Deng, Zhongkun Wang, Zhijun Zhang

**Affiliations:** 1 School of Civil Engineering, Guizhou Institute of Technology, Guiyang 550003, China; jfan1988@163.com (J.F.); dengsijie1988@163.com (S.D.); chengweideng1@163.com (C.D.); zhijun0973@163.com (Z.Z.); 2 College of Water Conservancy and Civil Engineering, South China Agricultural University, Guangzhou 510642, China; 13526578601@163.com

**Keywords:** carbon nanotube, styrene-acrylic emulsion, cement paste, mechanical properties, microstructure

## Abstract

Carbon nanotubes (CNTs) are considered as one of the ideal modifiers of cement materials, since CNTs can improve the mechanical properties of cement paste effectively. However, the interfacial interaction between CNTs and the cement matrix is weak. Moreover, it is difficult to disperse CNTs within cement paste. To overcome these shortages, in this study, CNTs were firstly dispersed into a styrene-acrylic emulsion (SAE). Then the homo-dispersion CNT/SAE emulsion was incorporated into cement paste. The effect of the CNT/SAE hybrid-system on the mechanical properties and microstructure of cement paste was studied. For purposes of comparison, the properties of cement paste mono incorporating CNTs or SAE are also investigated. The results show that CNT/SAE network films could be observed in cement paste by using a field emission scanning electron microscope (FESEM). These network films could bridge the cracks and refine the pores of a cement matrix. Infrared analysis and Raman spectroscopy show that the CNT/SAE hybrid modifier has stronger interfacial adhesion and better load transfer ability over the mono adding of CNTs and SAE emulsion. As a result, the hybrid addition of CNT/SAE significantly improved the flexural strength of cement paste. Especially, the addition of 0.1% CNTs and 15% SAE by mass of cement improved the 28-day flexural strength of cement paste by 21% and 25% as compared to the mono addition of CNTs or SAE, respectively.

## 1. Introduction

Cementitious materials are the most widely used building materials with high brittleness. The brittleness characteristics make cement-based material prone to crack under the action of external force, leading to the mechanical properties and durability of cement material decrease [1,2,3]. Fiber reinforcement is regarded as a typical and effective method to control cracking in cement-based materials. However, fibers in cementitious materials can delay the development of microcracks but they do not stop their formation [4].

The use of carbon nanotubes (CNTs) as a modifying material is an effective method to improve the mechanical properties, which may prevent the formation of micro-cracks in cementitious materials. This is due to the crack-bridging effect and pore-filling effect of CNTs [5,6,7]. In addition, the incorporation of CNTs can play a certain role in improving the durability of cement-based materials (drying shrinkage, chloride corrosion, etc.) [8,9]. Konsta-Gdoutos et al. [10] found that the addition of 0.1% CNTs (in weight percentage of cement, same as below) improve the flexural strength, Young’s modulus and the corrosion resistance of cement mortar. Weiwen Li et al. [11] found that the addition of 0.3% CNTs in weight percentage of cement increase the flexural and compressive strength of cement mortar by about 23% and 21%, respectively. Moreover, and under freeze-thaw cycles, the strength loss of cement specimens was much lower than the reference sample.

Although the beneficial effects of CNTs in cementitious materials have been demonstrated by the above studies, some problems associated with their use have yet to be solved. CNTs have high aspect ratios, and the strong van der Waals attraction among the nanotubes leads to the formation of bundles. To improve the dispersion of CNTs in cementitious materials, the common approach is to employ a sonication process with a surfactant. However, it was found that the applied surfactant often acts as an air entraining agent in cementitious materials and degrades the mechanical properties of the cement composites [12,13]. Even if CNTs are well dispersed in the mix water, another problem called geometric clusters will still prevent uniform dispersion in the cement matrix. Geometric clusters will occur when the cement grains in the composite are much larger than the distance between the inclusions. At complete hydration, the cement matrix comprises hydration products, void space and CNTs. The CNTs mainly distribute in the regions originally occupied by the water. In addition, another major challenge is related to how to bridge the big cracks of the cement matrix [14]. The mechanical behavior of the cement matrix is governed by the formation and propagation of cracks among the hydration products, and the small size of the nanotubes makes them ineffective in bridging and controlling these cracks propagation. It would be ideal if there was a way to “link” CNTs together into a longer chain so they can exhibit a stronger stress-transferring capacity from tubes to cementitious matrix.

Recent studies have shown that the use of polymer latex as a modifying material is an effective method to improve the flexural strength and impermeability of cementitious materials [15]. The addition of latex results in the formation of polymer films inside the cement matrix, leading to higher tensile and flexural strengths [16,17] and lower water absorptivity and chloride diffusivity [18]. With these improved properties, polymer-modified cement materials have been widely used in bridge decks, water proofing of roof decks, anti-corrosive linings under various chemical environments and grouts for concrete repair. In addition, the polymer side chain has a large number of active groups. The combination of chemical and physical dispersal can evenly disperse CNTs and form perfect crystal nuclei [19,20].

In this study, styrene-acrylic emulsion (SAE) was used as polymer latex. SAE has good adhesion performance, deformation adaptability, water resistance and salt corrosion resistance. Using it as a modified material in cement concrete can effectively improve its bonding ability, flexural strength and resistance to chloride ion penetration [21,22]. In addition, previous studies have shown that the hybrid of SAE and fiber could further improve the bending performance and tensile deformation characteristics of cement-based materials [23].

Based on the excellent performance of CNT and SAE as mentioned above, in this paper, SAE is used to disperse CNTs as well as to improve the bonding force between CNTs and cement matrix; as a result, the enhancement in the mechanical properties and microstructure of cement paste would be expected. The compressive strength, flexural strength and capillary water absorption of cement pastes modified by CNT/SAE were tested and compared with those of pure cement paste, the cement paste containing CNTs or SAE alone. The microstructures were also studied by using SEM, mercury intrusion porosimetry (MIP), FTIR and Raman spectroscopy to understand the mechanisms behind the performance of cement paste with and without modifier.

## 2. Materials and Methods

### 2.1. Materials

The cement used in this paper was P. O 42.5 R Portland cement (produced by Guangdong Tapai Cement Co. Ltd.). The chemical properties of cement provided by the manufacturer are listed in Table 1. The SAE latex with a solids content of 48 wt.% and viscosity of 500–2000 Pa·s at 23 °C, supplied by Shanghai Baolijia New Material Co. Ltd., China, was used. The physicochemical characteristics of SAE latex provided by the manufacturer are given in Table 2. The defoaming agent was produced by Guangdong Defeng Chemical Industry Co. Ltd. in China. Two different types of multi-walled carbon nanotubes, ordinary CNTs (without surface functionalization) and hydroxyl CNTs (with –OH functional groups, CNT–OH), provided by Chengdu Organic Chemicals Co. (China), were used in this study. The physical and chemical properties of CNTs provided by the manufacturer are shown in Table 3. The price of CNTs is about 100~150 USD per kg, the adding of CNTs may increase the cost of cement materials greatly. To overcome this shortage, the industrial-grade CNTs are used in this paper. At the same time, only 0.1% of CNTs in weight of cement are added.

### 2.2. Sample Preparation and Mix Design

Five mixes were prepared to investigate the mechanical properties and microstructure of cement paste. The details of the mix proportions are given in Table 4, where the control sample (PC) is pure cement paste without any modifier, HPC is cement paste incorporating 0.1 wt.% CNTs-OH alone, SPC only contains 15 wt.% SAE, NSPC includes 0.1 wt.% CNTs and 15 wt.% SAE; HSPC contains 0.1 wt.% CNTs-OH and 15 wt.% SAE. The water-cement ratio was adjusted by maintaining the same flow table value (because polymer latex is beneficial for reducing the water demand of cement [21]).

**Figure 1:**
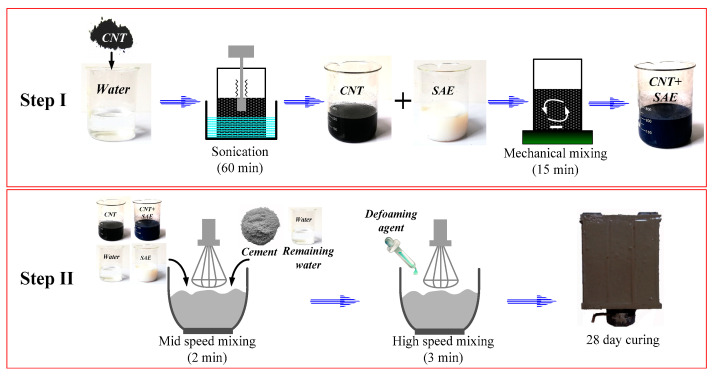
Flow chart of the preparation of the samples.

The specimens for NSPC and HSPC (hybrid containing SAE and CNTs or CNTs–OH) were prepared using the following steps, and the procedure is also summarized in Figure 1:

(1) The weighed quantity of CNT-powder (CNTs or CNTs–OH) was dissolved in deionized water and evenly stirred by using KQ410HT ultrasonic vibration (40 kHz and 200 W);

(2) Adding CNTs into the prepared SAE latex and mixing by magnetic stirring (FK-H1) for 15 min at 60 °C with a rotation speed of 2000 rpm to achieve a uniform distribution of nano-materials.

(3) Adding hybrid CNT/SAE mixture, water, superplasticizers and defoamers (at the amount of 0.14 wt.%) into the above cement paste mixture and mixing for another 5 min;

(4) Pouring the mix into oiled molds and using an electric vibrator to ensure good compaction;

(5) Smoothening the surfaces of specimens and then covering them with wet clothes;

(6) Demolding the specimens after 24-h curing and then curing them in air under room temperature for 28 days (watering every day for the first 7 days).

To prepare specimens for HPC, Step (2) was not necessary, and Step (1) was changed to: Adding CNTs–OH and superplasticizers (as surfactant) into water and mixing by using ultrasonic vibration for 1.5 h at 60 °C to make suspension with uniform distribution of nano-materials.

### 2.3. Testing Methods

**Figure 2:**
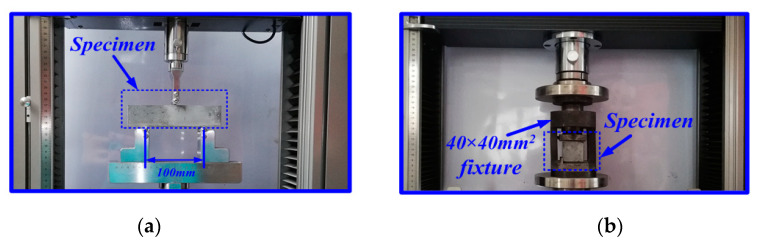
Loading setup of the flexural strength (**a**) and compressive strength tests (**b**).

The flexural and compressive strength of cement composites were performed in accordance to GB/T 17671-1999 [24]. The CMT-5105 universal testing machine produced by Shenzhen Xinsansi Co. Ltd. China was used for the strength measurement. The flexural strength of cement composites was tested after 3, 7 and 28 days curing by using a three-point bending method with a span of 100 mm and a loading speed of 50 ± 10 N/s, as shown in Figure 2a. For each mix, three samples were tested, and the average value was used as the flexural strength. After the flexural strength testing, six broken pieces of each mix were used to determine the compressive strength, the test size was 40 × 40 mm, as shown in Figure 2b, and the loading rate was 2400 ± 200 N/s.

**Figure 3:**
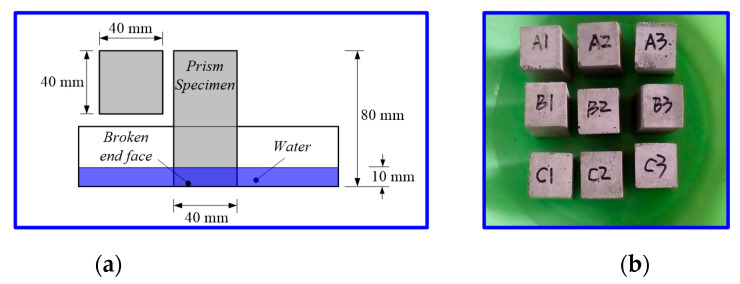
Schematic (**a**) and example image (**b**) of the capillary water absorption test setup.

The capillary water absorption of cement composites after the 28-day curing period was carried out according to DIN 52617 [25]. For each mix, three samples with a size of 40 × 40 × 80 mm were dried in a blast air oven for 48 ± 2 h at 105 ± 2 °C. After drying, the samples were removed from the oven, cooled in an air-tight container and then the four sides were sealed by using a layer of wax. Each specimen was then weighed and immediately immersed in water with a depth of 10 mm, ensuring, as shown in Figure 3, to keep the untreated surface in the vertical direction. After removing from water, the surface of the specimen was wiped and the specimen was weighed again. The water absorption at a given time was calculated from the increase in mass of the specimen and expressed.

Raman spectroscopy has become one of the most important techniques for analyzing carbon composites [26,27,28]. The Raman spectrum of the CNTs was measured by HR800 Raman spectroscopy produced by HORIBA, France. During the test, a solid-state crystal laser with a wavelength of λ = 532 nm was selected as the laser emission source. Before testing, each sample was grinded into a fine powder passing through a 1000 mesh sieve, the diameter of sample was approximately 7 μm, the confocal hole was 500 μm and the beam intensity was less than 1 a.u.

The morphology and distribution characteristics of ordinary CNTs and CNTs–OH were observed by using a JEM-2100F transmission electron microscope (TEM, 200 kV). Before testing, CNTs were added into water and sonicated for 60 min (40 kHz and 200 W). The test voltage was 200 kV.

The porosity and pore size distribution are considered to be one of key factors that affect the mechanical properties and durability of cement-based materials. In this test, the porosity and pore size distribution of cement paste with different mix proportions were tested by using an AutoPore 9500 mercury indenter manufactured by Micromeritics in the United States. The samples were dried in an oven at 60 ± 2 °C for 48 h before testing.

Infrared analysis was carried out by using the MAGNA-IR750 spectrophotometer produced by Nichols, USA. Before testing, the sample was ground and mixed with KBr powder. Then, the sample was placed on a tablet press, and then sent to the sampling chamber. The wavenumber range was 500 to 4000 cm^−1^.

The microstructure of cement composites was observed by using the Hitachi SU4800 field emission scanning electron microscope (FESEM). Before testing, the sample was vacuumed, and then placed in the observation room of FESEM for observation. The topography of the representative area was selected for photographing. The sample was observed with an acceleration voltage of 5.0 kV.

## 3. Results and Discussion

### 3.1. Carbon Nanotube-Raman Spectra and Transmission Electron Microscopy

**Figure 4:**
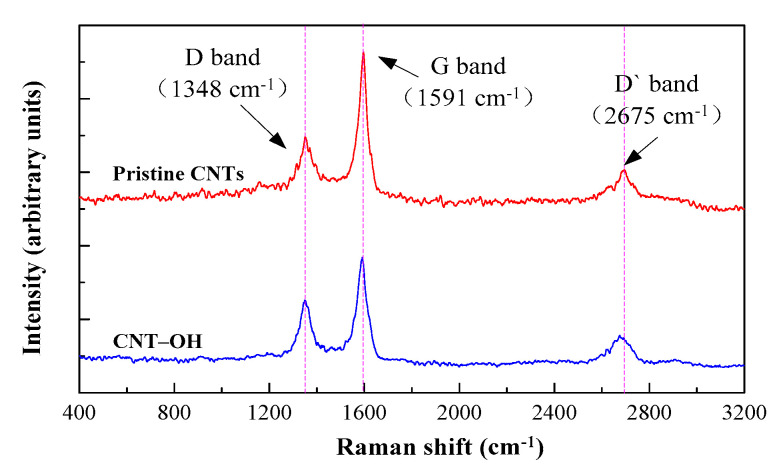
Raman spectra from carbon nanotubes.

Figure 4 shows the 532 nm excited Raman spectra of neat CNTs and CNT–OH. The Raman spectrum of the neat CNTs and CNT–OH displays three typical bands with intensity maxima of approximately 1348 cm^−1^ (D-band), 1591 cm^−1^ (G-band) and at ~2675 cm^−1^ (G’-band). The D-band at approximately 1348 cm^−1^ originates from sp^2^ hybridization in the graphitic structure. This Raman peak is the most sensitive to nanotube alignment. The G-band at 1571 cm^−1^ is attributed to the in-plane vibrations of the graphitic wall. The G’ peak reflects the efficiency of stress transfer along the interface between the CNTs and matrix [29]. Furthermore, these bands are associated with defects and doping and; therefore, they can be used to characterize and monitor the structural changes in CNTs [30]. The ratio I_G_/I_D_, the intensity ratio between the D band (I_D_) and G band (I_G_), has been used as the quality indicator of carbon-based systems. Generally, the less disordered a carbon-based system is, the lower the ratio I_D_/I_G_ is expected to be [31]. For the spectrum shown in Figure 4, the I_G_/I_D_ ratio of CNT–OH is almost 0.69, while for the original CNT, the ratio is about 0.56, indicating the existence of a higher order of defects in the functionalized CNT. These defects play a key role in improving the dispersion of CNTs in cement paste.

**Figure 5:**
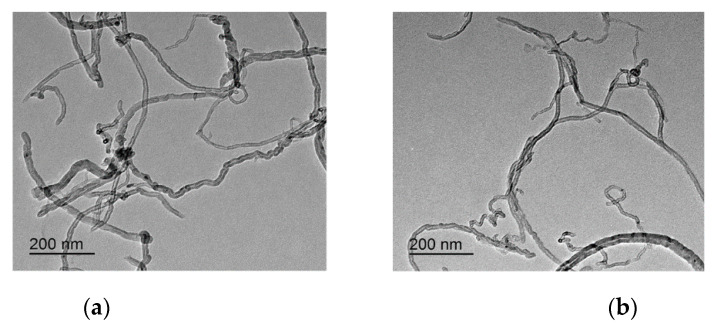
TEM micrographs of neat CNTs (**a**) and CNTs–OH (**b**) dispersed in water.

The morphology and distribution of the two types of carbon nanotubes were further observed by TEM, as shown in Figure 5. The neat CNTs remained agglomerated in water after 60 min of ultrasonic dispersion, and these aggregates could easily be observed with the naked eye because they precipitated quickly (Figure 5a). As shown in Figure 5b, CNT–OH was more uniformly dispersed in water over CNTs with the aid of ultrasonication, indicating the hydrophilic functional groups of CNT–OH enhance its compatibility with water.

### 3.2. Mechanical Properties of Cement Composites

**Figure 6:**
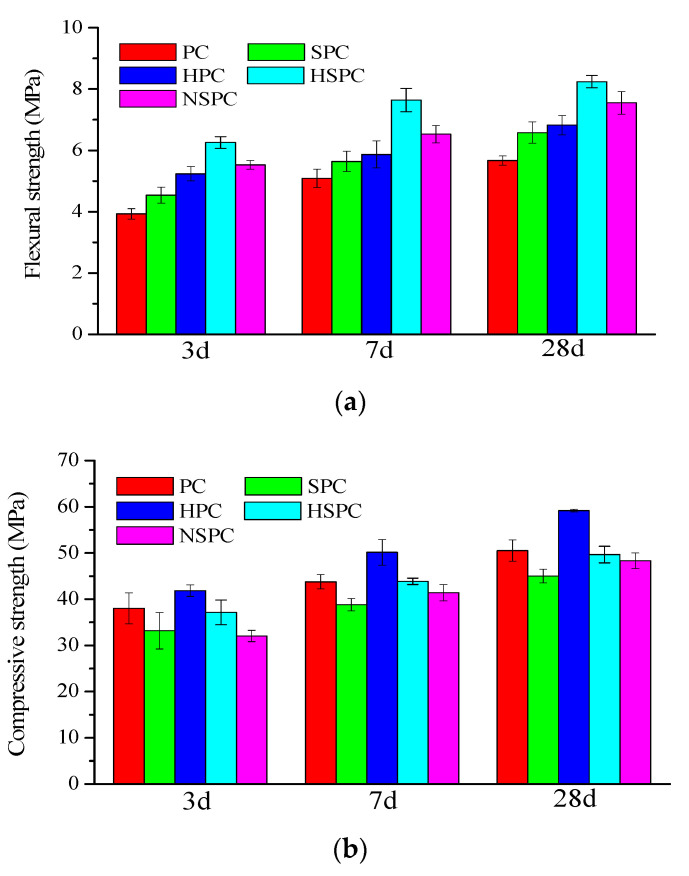
Flexural strength (**a**) and compressive strength (**b**) of cement composites after 3, 7 and 28 days of curing.

The effects of CNTs and SAE on the mechanical properties of cement paste were investigated, and the 3-, 7-, and 28-day flexural and compressive strengths are presented in Figure 6. It can be found that the addition of CNT–OH (0.1 wt.%) could increase the flexural and compressive strengths of cement paste. As compared to the reference group(PC), the compressive strength of HPC increased by 10%, 14% and 17% after 3, 7 and 28 days curing, respectively. At the same time, the flexural strength of cement paste increased by 33%, 15% and 20%, respectively. The enhancement of CNTs on the mechanical property of cement paste could be attributed to the pore-filling and crack-bridging effects of CNTs, as stated by researchers [32]. Moreover, as shown in Figure 6, the incorporation of 15% SAE by weight percentage of cement could increase the 3-, 7-, and 28-day flexural strength by 15%, 11% and 12% as compared to PC, respectively. In regards to the compressive strength, the incorporation of 15% SAE leads to a decrease of 14%, 14% and 12% as compared to PC after 3, 7 and 28 days curing, respectively. This was consistent with the test results found in [21]. The combination of CNTs and SAE into a hybrid solution enhanced the mechanical properties of cement paste greatly. Especially, HSPC had the highest flexural strength at given ages. This enhancement may be ascribed to the hydrophilic group –OH on the surface of CNT–OH, which not only promotes the dispersion of CNT particles in the aqueous solution but also enhances the bonding behavior among cement hydration, SAE and CNTs–OH. As shown in Figure 6, the flexural strength of HSPC was 59%, 50% and 45% higher than that of PC after 3, 7 and 28 days curing, respectively. Additionally, Figure 6 shows that the 3-, 7- and 28-day flexural strength of HSPC were about 19%, 30% and 21% higher than that of HPC, respectively. This may indicate that SAE can “link” CNTs–OH together into a longer chain so they can exhibit a stronger stress-transferring capacity. As compared with SPC, the hybrid combining SAE and CNTs–OH increased the 3-, 7- and 28-day flexural strength by 38%, 35% and 25%, respectively, implying CNTs–OH enhance the ultimate bearing capacity of the SAE polymer. Moreover, Figure 6 shows that the hybrid incorporating ordinary CNTs and SAE (NSPC) increased the 3-, 7- and 28-day flexural strength of cement paste by 41%, 28% and 33% as compared to PC, respectively. As compared to the HPC and SPC groups, the 3-, 7- and 28-day flexural strength of NSPC increased by 6%, 11% and 11% and 22%, 16% and 15%, respectively.

### 3.3. Capillary Water Absorption of the Cement Composites

**Figure 7:**
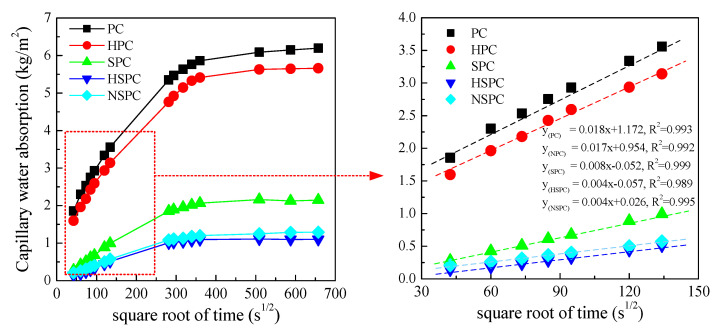
Capillary water absorption of the cement composites versus the square root of time.

Figure 7 shows the relationship between the cumulative water absorption rate (infiltration mass per unit area) and the square root (t^1/2^) of test time. The slope of the absorption rate curve was defined as the capillary water absorption coefficient; and the higher the absorption coefficient, the faster the water absorption rate will be. Figure 7 shows that the absorption curve shapes of the five test samples were quite similar, where the water absorption rate increased fast at the early testing time, and then tended to be gently. However, the water absorption mass and absorption coefficient varied with the addition of modifier greatly. Clearly, according to Figure 7, the reference PC group had the highest water absorption rate and sorptivity coefficient. With the addition of CNTs, the water absorption characteristics of the composites exhibited a slight improvement because of the filling effect of the micropores. Compared with the PC group, the water absorption rate (120 h) and sorptivity of the HPC were reduced by 9% and 6%, respectively. In addition, from the test results it can be seen that, with the addition of the polymer latex, the capillary absorption rate of the cement matrix decreased substantially, which was consistent with the results of [21]. The capillary water absorption rate (120 h) and sorptivity of the SPC group were 2.15 kg/m^2^ and 0.008 kg/s^1/2^, which were 65% and 55% lower than those of the PC group, respectively. The reasons for the low water capillary adsorption could be attributed to the sealing effect of the polymer film formed in the cement matrix [33]. When the cementitious composite contained both CNTs and SAE, the water adsorption was further reduced with the synergistic hole filling effect of the two materials, especially for the addition of functionalized CNTs (HSPC group). The HSPC group had the lowest coefficient rate (120 h) and sorptivity, which were 1.10 kg/m^2^ and 0.004 kg/s^1/2^ and were 82% and 78% lower than that of the PC, respectively. This indicates that the simultaneous incorporation of CNTs and SAE latex significantly improved the water permeability resistance of cement paste.

### 3.4. Porosity and Pore Size Distribution of Cement Pastes

**Figure 8:**
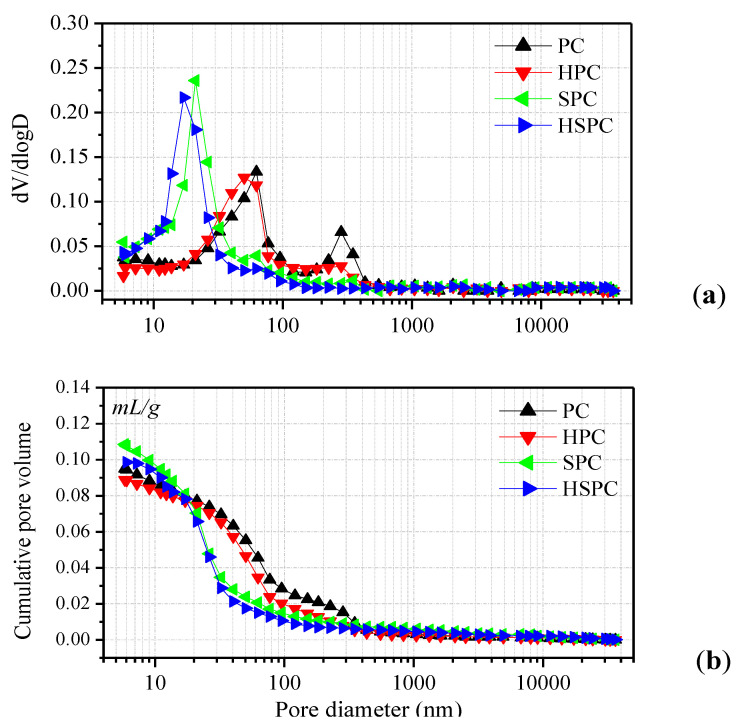
Relation between pore volume (**a**) and cumulative pore volume (**b**) with respect to pore size for composites.

The porosity and pore size distribution are important indicators for evaluating the function and performance of cement-based materials. When the porosity and pore size increase, the mechanical properties and durability of cement material decrease. According to [34], the pore size of 50 nm is a critical value to differentiate micro- and macro-capillary pores. Pores larger than 50 nm are known as the macro-capillary pores which determine the strength and impermeability characteristics, while the pores smaller than 50 nm are micro-capillary pores which mainly impact the creep and dry shrinkage of cementitious materials. Mercury intrusion porosimetry (MIP) is used to test the pore size distribution and porosity cement paste with or without modifier after 28 days curing, and the results are shown in Figure 8. The value of dV/dlogD means the ratio of the pore volume at a certain diameter to the total pore volume. For PC, there are two dV/dlogD peaks in the curve: The first peak value of 0.068 is located at the 280 nm diameter, and the second peak value of 0.136 is at the 60 nm zone. For the HPC group, two dV/dlogD peaks could be found on the MIP curve, but the corresponding pore size and ratio are reduced (values of 0.026 at 280 nm and 0.125 at 50 nm) as compared to PC, indicating the pore-filling effect of CNT–OH. For the SPC group, with 15% SAE incorporated, the dV/dlogD peak of 0.239 is located at a much lower diameter of 20 nm over PC, no peak could be found on the diameter larger than 50 nm. This indicates that SAE has a more obvious effect on refining the pore size of cement paste. In addition, the dV/dlogD peak of HSPC is located at the lowest diameter of 15 nm, as shown in Figure 8, indicating the hybrid incorporation of CNT–OH and SAE could further refine the pore size of cement paste.

Figure 8b shows the porosity distributions of the composites, and the test results are summarized in Table 5. It can be observed that the total porosity and average pore size of the cement paste were slightly reduced with the addition of the CNT–OH. However, with the addition of SAE, the total porosity of the composites slightly increased, while the average pore size decreased substantially. In addition, the average pore size of the composites was further reduced when CNT–OH and SAE were compounded. The average pore size of the HSPC group was only 20.3 nm, which was 66% lower than that of the PC group (60 nm). This further illustrates that the CNT–OH/SAE system is effective in refining the pore size of cement composites.

### 3.5. FTIR Patterns of the Cement Pastes

**Figure 9:**
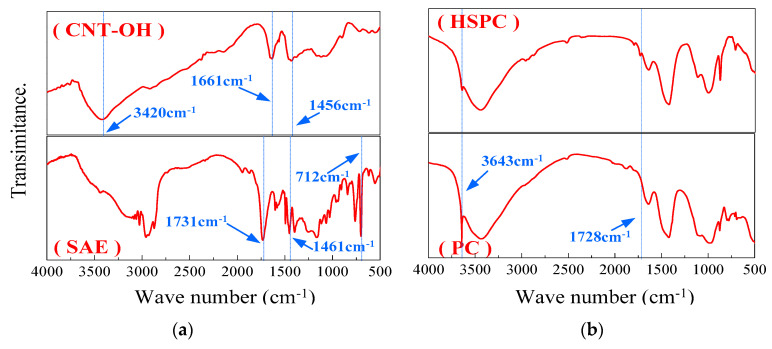
FTIR spectra of CNT–OH, SAE (**a**) and HSPC, PC (**b**) at 28 days.

The FTIR patterns of CNT–OH and SAE are shown in Figure 9a. There are three obvious absorption peaks in the FTIR curve of the CNT–OH samples at 3420, 1661 and 1456 cm^−1^, in which the absorption peak obtained at 3420 cm^−1^ is attributed to OH stretching vibrations of absorption water, the peak at approximately 1661 cm^−1^ corresponds to the C=C stretching vibrations and the band at 1456 cm^−1^ is due to the vibration of C=O [35]. The FTIR spectrum of pure SAE particles has three obvious absorption peaks at 1731, 1461 and 712 cm^−1^. The absorption peaks appear at 712 and 1461 cm^−1^ due to the vibration of the benzene ring skeleton, and the peak at approximately 1731 cm^−1^ is caused by the vibration of the carbonyl group. The FTIR spectra of the PC group (without CNT–OH and SAE) and HSPC group (with 0.1% CNT–OH and 15% SAE) are shown in Figure 9b. It can be clearly seen from the test results that the FTIR curves of the PC and HSPC both have four obvious absorption peaks at 3643, 3441, 1645 and 971 cm^−1^. The absorption peak at approximately 3643 cm^−1^ corresponds to the OH in the Ca(OH)_2_, and the absorption peak at 971 cm^−1^ is due to the Si–O vibration of the hydration product C–S–H gel. The absorption peaks appearing at 1646 and 3441 cm^−1^ are from the stretching of the O–H bonds in the hydration product. However, compared with the PC group, there is an additional absorption peak near 1728 cm^−1^ for HSPC that is generated by the vibration of the carbonyl group in the SAE powder. The same absorption peak also appears at 1731 cm^−1^ for the pure SAE powder in Figure 9a, and the peak drifts from 1731 to 1728 cm^−1^. In addition, after comparing the two groups of FTIR curves in Figure 9b, it can be found that the peak intensity of the HSPC at 3643 cm^−1^ is substantially lower than that of the PC group. The change in the absorption peak at 3643 cm^−1^ can be attributed to the complexation reaction between the SAE and Ca^2+^, which leads to a decrease in Ca(OH)_2_. This phenomenon has also been reported in [36]. The effect of CNT–OH on the absorption peak of the cement paste was not found.

### 3.6. Scanning Electron Micrographs of the Cement Pastes

**Figure 10:**
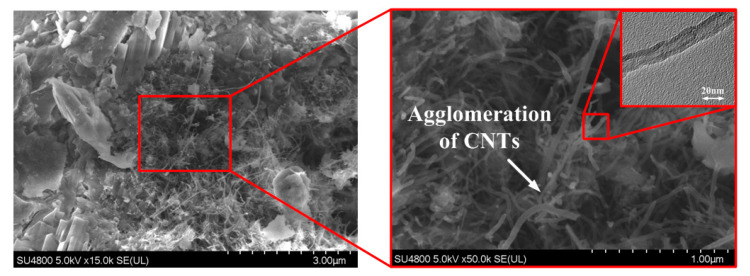
SEM images taken from the 28 day HPC hardened paste samples.

The morphology and microstructure, especially the defects (pores and cracks), are considered to be the main factors controlling the performance of cementitious materials. To better understand the effect of the CNT–OH/SAE on the properties of cement composites, the microstructures of the HPC, SPC and HSPC samples were observed under FESEM. Figure 10 shows the FESEM images of the HPC (with 0.1 wt.% CNT–OH) hardened paste samples at 28 days of hydration. In Figure 10, C–S–H gel, CNTs, pores and cracks can be easily found, and the microstructure of the cement matrix is slightly improved by the filling effect of the CNTs. However, it is clearly observed that the CNTs between the hydration products are extensive entanglements and agglomerations, although they are analyzed ultrasound technology for 60 min. In addition, CNTs are mostly distributed in the middle region, while the content of the nanoparticles is lower in the other regions. There are still many unfilled pores and cracks in the cement matrix, which indicates that the CNTs cannot be uniformly dispersed in the cement paste when treated by ultra-sonication alone. The non-uniformly dispersed CNTs limited the improvement of the mechanical properties of the cement composites.

**Figure 11:**
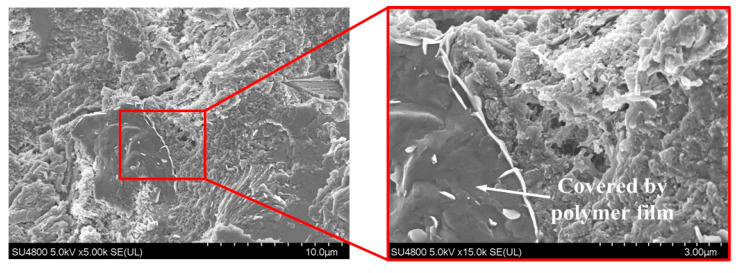
SEM images of the hardened SPC at 28 days.

Figure 11 presents the morphology of the cement paste incorporating SAE alone. Figure 11 clearly shows that the film formed by the SAE particles is wrapped on the surface of the hydration products, making the surface of the sample very smooth. In addition, it can also be observed that part of the polymer film is entangled with the main hydration product (such as ettringite) to form a dense crystal structure [22]. These polymer films are conducive to enhancing the flexural strength and water adsorption rate of cement composites (as shown in the results of Section 3.2 and Section 3.3). Obviously, when compared with the hydration products, the SAE film with an irregular shape shows a higher flexibility and strong cohesive force. In contrast to the CNT–OH (Figure 10), the SAE film seems to have a larger width and better viscidity. However, it should be noted that, in the test results shown in Figure 11, there are still obvious pores and cracks in the sample.

**Figure 12:**
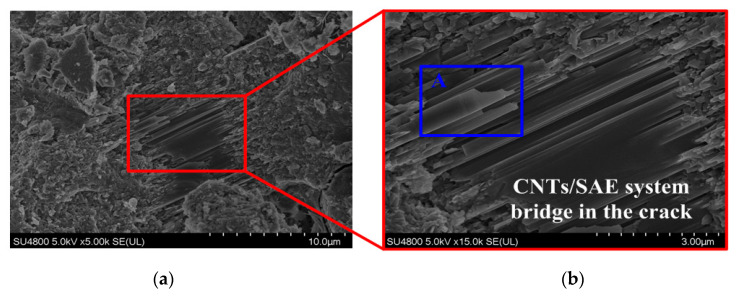

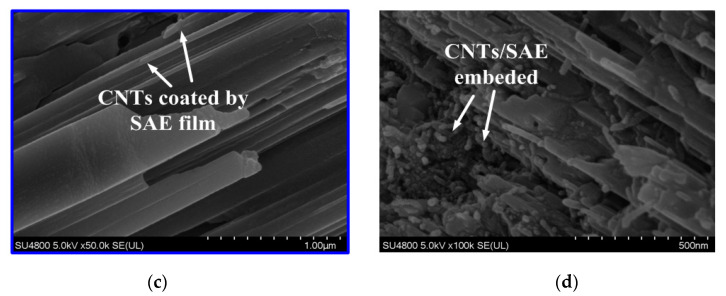
SEM micrographs of the (**a**) hardened HSPC; (**b**) magnified SEM image of the box region in (a); (**c**) magnification of point A in (b); and (**d**) CNTs/SAE embedded in the hydration products (for 28 days).

Figure 12 shows the defects of the HSPC incorporating 0.1 wt.% CNT–OH and 15 wt.% SAE after 28 days of curing. Figure 12a,b clearly reveals the interaction of the SAE and CNTs. The CNTs are coated by an SAE film, and CNT/SAE networks bridge over the cracks and fill the pores, leading to a dense microstructure. Compared with the HPC (Figure 10) and SPC (Figure 11) groups, the morphology of the hardened HSPC shows fewer pores and cracks. The formation of abundant CNT/SAE networks and the dense microstructure of HSPC can probably improve the toughness of cement paste, further study is worthy to be conducted. In addition, Figure 12c (enlargement of point A in Figure 12b) shows that, as compared with HPC (Figure 10), the distribution of CNTs was more uniform and fewer entanglements and agglomerations could be found in HSPC. Moreover, from Figure 12c, it can be found that CNTs coated by polymer film adhere tightly to the cement matrix, indicating the strong interaction among CNTs, SAE and cement hydration products. Furthermore, it can be seen from Figure 12d that some CNTs/SAE networks are embedded in cement matrix, leading to the reduction of the pore size as demonstrated by the MIP test, as shown Figure 8 and Table 5. In short, the cement paste incorporating the CNTs/SAE hybrid solution had a more uniform and denser microstructure than that incorporating CNTs or SAE alone, thereby, the hybrid addition of CNTs and SAE is more effective in enhancing the flexural strength and water resistance of cement composites, as shown in Figure 6 and Figure 7.

## 4. Conclusions

This study systematically analyzed the mono and hybrid effects of ordinary carbon nanotubes (CNTs) or hydroxyl carbon nanotubes (CNTs–OH) and styrene acrylic emulsion (SAE) on the mechanical performance, water absorption and the microstructure of cement paste. According to the test results in this study, the main achievements were:

i. The mechanical properties of the cement composites show that the combined use of 0.1 wt.% CNT–OH and 15 wt.% SAE in the cement matrix can increase the flexural strength by approximately 45% at 28 days and was obviously higher than that of the addition of CNT–OH or SAE alone.
ii. The capillary water absorption test results show that the simultaneous incorporation of CNTs and SAE latex can significantly improve the water permeability resistance of cement paste. The water absorption rate (120 h) and sorptivity of HSPC were 82% and 78% lower than that of the PC, respectively.
iii. The mono and hybrid additions of CNTs–OH and SAE could decrease the porosity and refine the pore structure of concrete, as demonstrated by the MIP analysis.
iv. According to FTIR analyses, the addition of SAE and CNTs–OH decreased the Ca(OH)_2_ content of cement paste, indicating the interaction takes place among SAE, CNTs–OH and cement hydration productions.
v. CNT–OH/SAE hybrid network films could be observed in cement paste by using FESEM. The crack-bridging and pore-filling effects of networks were responsible for the enhancement of the CNT–OH and SAE hybrid solution on the mechanical properties and water resistance of cement paste.

## Figures and Tables

**Table 1:** Chemical composition of the Portland cement.

**P. O 42.5 R**	**Raw Material (%)**								
	SiO_2_	Al_2_O_3_	CaO	MgO	Na_2_O	K_2_O	Fe_2_O_3_	SO_3_	Loss on ignition
	18.3	4.5	62.4	2.1	0.3	1.5	2.3	3.5	2.6

**Table 2:** Physicochemical characteristics of the SAE latex.

**SAE Latex**	**Solid Content**	**T_g_**	**Viscosity**	**pH**	**Minimum Film Forming Temperature**	**Appearance**
	48 ± 1 (wt.%)	23 (°C)	500–2000 (mPa·s)	7~9	21 (°C)	Milky white liquid

**Table 3:** Properties of carbon nanotubes (CNTs).

Raw Material	External Diameter (nm)	Purity (wt.%)	Length (μm)	Specific Surface Area (m^2^/g)	ASH (wt%)	Tap Density (g/cm^3^)	–OH Content (%)
**CNT**	10~30	95	10~30	>110	<5	0.14	–
**CNT–OH**	10~30	95	10~30	>110	<5	0.14	2.48

**Table 4:** Mix proportions of the composites.

Samples ID	Ratio of Material Mass to Cement Mass (%)					m_w_/m_c_	Flow/mm
	Cement	Water	CNT	SAE	Defoamer		
**PC**	100	40	0	0	0.14	0.4	178
**HPC**	100	40.04	0.1 (CNTs–OH)	0	0.14	0.4	176
**SPC**	100	32	0	15	0.14	0.32	182
**NSPC**	100	32.03	0.1 (CNTs)	15	0.14	0.32	179
**HSPC**	100	32.03	0.1 (CNTs–OH)	15	0.14	0.32	181

**Table 5:** Pore size distribution of composites.

Samples ID	Total Intruded Volume (mL/g)	Total Porosity (%)	Average Pore Diameter (nm)
**PC**	0.0950	16.64	60
**HPC**	0.0889	16.30	52.3
**SPC**	0.1085	18.71	24.5
**HSPC**	0.0986	17.11	20.3
